# Relationships Between Leaf Carbon and Macronutrients Across Woody Species and Forest Ecosystems Highlight How Carbon Is Allocated to Leaf Structural Function

**DOI:** 10.3389/fpls.2021.674932

**Published:** 2021-06-11

**Authors:** Kaixiong Xing, Mingfei Zhao, Ülo Niinemets, Shuli Niu, Jing Tian, Yuan Jiang, Han Y. H. Chen, Philip J. White, Dali Guo, Zeqing Ma

**Affiliations:** ^1^Key Laboratory of Ecosystem Network Observation and Modeling, Center for Forest Ecosystem Studies and Qianyanzhou Ecological Station, Institute of Geographic Sciences and Natural Resources Research, Chinese Academy of Sciences, Beijing, China; ^2^State Key Laboratory of Earth Surface Processes and Resource Ecology, Faculty of Geographical Science, Beijing Normal University, Beijing, China; ^3^Institute of Agricultural and Environmental Sciences, Estonian University of Life Sciences, Tartu, Estonia; ^4^Estonian Academy of Sciences, Tallinn, Estonia; ^5^Key Laboratory of Plant-Soil Interactions, Ministry of Education, College of Resources and Environmental Sciences, China Agricultural University, National Academy of Agriculture Green Development, Beijing, China; ^6^College of Natural Resources Management, Lakehead University, Thunder Bay, ON, Canada; ^7^College of Geographical Sciences, Fujian Normal University, Fujian, China; ^8^The James Hutton Institute, Dundee, United Kingdom; ^9^Zoology Department, College of Science, King Saud University, Riyadh, Saudi Arabia

**Keywords:** stoichiometry, leaf carbon concentration, leaf calcium concentration, leaf structural strategies, cell wall composition, leaf cellular structure

## Abstract

Stoichiometry of leaf macronutrients can provide insight into the tradeoffs between leaf structural and metabolic investments. Structural carbon (C) in cell walls is contained in lignin and polysaccharides (cellulose, hemicellulose, and pectins). Much of leaf calcium (Ca) and a fraction of magnesium (Mg) were further bounded with cell wall pectins. The macronutrients phosphorus (P), potassium (K), and nitrogen (N) are primarily involved in cell metabolic functions. There is limited information on the functional interrelations among leaf C and macronutrients, and the functional dimensions characterizing the leaf structural and metabolic tradeoffs are not widely appreciated. We investigated the relationships between leaf C and macronutrient (N, P, K, Ca, Mg) concentrations in two widespread broad-leaved deciduous woody species *Quercus wutaishanica* (90 individuals) and *Betula platyphylla* (47 individuals), and further tested the generality of the observed relationships in 222 woody eudicots from 15 forest ecosystems. In a subsample of 20 broad-leaved species, we also analyzed the relationships among C, Ca, lignin, and pectin concentrations in leaf cell walls. We found a significant leaf C–Ca tradeoff operating within and across species and across ecosystems. This basic relationship was explained by variations in the share of cell wall lignin and pectin investments at the cell scale. The C–Ca tradeoffs were mainly driven by soil pH and mean annual temperature and precipitation, suggesting that leaves were more economically built with less C and more Ca as soil pH increased and at lower temperature and lower precipitation. However, we did not detect consistent patterns among C–N, and C–Mg at different levels of biological organization, suggesting substantial plasticity in N and Mg distribution among cell organelles and cell protoplast and cell wall. We observed two major axes of macronutrient differentiation: the cell-wall structural axis consisting of protein-free C and Ca and the protoplasm metabolic axis consisting of P and K, underscoring the decoupling of structural and metabolic elements inherently linked with cell wall from protoplasm investment strategies. We conclude that the tradeoffs between leaf C and Ca highlight how carbon is allocated to leaf structural function and suggest that this might indicate biogeochemical niche differentiation of species.

## Introduction

Recent studies on macronutrient stoichiometry have improved the understanding of the relationships between plant structure and function and demonstrated how leaf physiological processes vary with environmental conditions (Maire et al., 2015; Tian et al., 2017; Yue et al., 2017; Zhang et al., 2018; Chaturvedi et al., 2021). Environmental conditions, such as light, temperature, soil nutrients, affect the acquisition of carbon (C), nitrogen (N), and phosphorous (P) and the macro element partitioning in plants (Hoch, 2007; Liu et al., 2013; Lena and Wim, 2018). However, in nutrient stoichiometry studies, relatively little attention has been paid to the relationships between C and other nutrients [e.g., calcium (Ca) and magnesium (Mg)] (McGroddy et al., 2004; Han et al., 2011; Hao et al., 2015). Although leaf C:N:P ratios under optimal conditions are relatively conserved worldwide (McGroddy et al., 2004), the presence of excess C-rich compounds in the structural components (e.g., lignin) and defense components (phenolics, terpenoids) results in higher C:nutrient ratios in higher plants (Hessen et al., 2004). Indeed, the combinations of various compounds with different C costs and a turnover rate ultimately impact the stoichiometry of leaf C constituents and ecosystem C cycle (Poorter et al., 1997; Ma et al., 2018). Thus, detailed elucidation of the determinants of leaf C concentration may provide informative insight into plant C allocation strategies.

Carbon is the critical structural and metabolic element with the highest concentration in leaf dry matter, and leaf C variations are indicative of differences in the distribution and utilization of photosynthetic products and different combinations of various compounds with different C costs (Hessen et al., 2004; McGroddy et al., 2004; Han et al., 2011). Structural C [*C*_S_: protein-free C concentration (Niinemets and Tamm, 2005)] is mainly related to the cell wall components that provide biomechanical support and protection for plant tissues. Woody angiosperms have developed similar major cell wall components during evolution (Popper, 2011). These components comprise lignin, cellulose, hemicellulose, and pectins (including protopectin and water-soluble pectin) (Kärkönen and Koutaniemi, 2010; Li et al., 2016; Boanares et al., 2018; Wang and Cosgrove, 2020). Several studies have observed a positive relationship between leaf C and lignin concentrations in the cell walls of plants (Niinemets, 1997; Villar et al., 2006; Kärkönen and Koutaniemi, 2010; Meents et al., 2018). However, C-related leaf structural strategies can vary among species, environments, and plant life forms. For example, leaf lignin accumulates, and cellulose concentration decreases under high irradiance and photosynthesis conditions (Niinemets and Kull, 1998; Poorter et al., 2006). In low-temperature environments with restricted photosynthesis, both lignin and cellulose concentrations decrease in leaves, whereas hemicellulose investment is not affected (Richardson, 2004; Hoch, 2007). Furthermore, evergreen broad-leaved trees invest more in lignin and phenols than deciduous trees, resulting in greater structural C concentrations (Poorter et al., 2006; Osunkoya et al., 2008).

Leaf stoichiometry of C and other key elements can provide insight into a tradeoff between structural and metabolic investments. First, N, P, and K are mainly partitioned to cell protoplasm and regulate the rate of principal physiological processes such as photosynthesis and synthesis of biomolecules (Hawkesford et al., 2012). Thus, the relationships among C–N, C–P, C–K can indicate variations in balancing investments in leaf structural and metabolic functions. Second, a leaf C-silicon (Si) tradeoff in grasses has been observed (Quigley et al., 2020). Like Si in grasses, Ca participates in cell wall construction in most angiosperms, except grasses (White and Broadley, 2003; Hawkesford et al., 2012; White et al., 2018). A considerable amount of leaf Ca is bound as Ca^2+^-pectate (*Pectate*_2_*Ca*_10_) in the middle lamella of the cell wall, whereas the proportion of cell wall Ca concentration can be up to 50% of total leaf Ca even in Ca-deficient soils (Hawkesford et al., 2012). Adequate Ca supply also enhances cellulose fiber growth, increases the thickness of secondary cell walls, improves cell wall load-bearing functions and aids cell wall rigidification (Conn et al., 2011; Peaucelle et al., 2012; Atmodjo et al., 2013; Wang and Cosgrove, 2020). Thus, it is reasonable to expect the existence of a leaf C–Ca tradeoff in angiosperms other than grasses. Third, 5–10% Mg in leaves is present as Mg^2+^-pectate in the middle lamella of cell walls (Hawkesford et al., 2012; White and Holland, 2018).

Overall, the leaf stoichiometric interrelationships among C, –Ca, –Mg, –P, –N, and –K can be used to infer the leaf construction cost and metabolic intensity (Meents et al., 2018; Haas et al., 2020). Fundamentally, leaf macronutrient stoichiometry and metabolic processes are affected by the share of investments in leaf structure and metabolic activity and by cellular structure, in particular the construction of cell walls. The cell wall consists of lignin, hemicellulose, cellulose, and Ca^2+^-pectates. The construction cost of lignin is 1.75- and 1.50-fold higher than that of pectins and (hemi-)cellulose, respectively (Poorter et al., 1997). In contrast, Ca^2+^-pectate is a much cheaper load-bearing component with the lowest C concentration for most non-graminoid angiosperms (18.8%) (Poorter et al., 1997; Niinemets and Kull, 1998; Proseus and Boyer, 2008; Osunkoya et al., 2010). Although cellulose networks are also involved with cell wall load-bearing capacity, lignin crosslinks and Ca^2+^-pectates complexes generally play a greater role (Peaucelle et al., 2012; Wolf et al., 2012). This is especially relevant for woody species that often experience low-leaf water potential below turgor loss points without wilting (Niinemets, 2001). Accordingly, the load-bearing capacity of woody species depends much less on turgor pressure and concentrations of ionic and neutral osmotic than the mechanical properties of leaves of herbaceous species. The evidence summarized collectively indicates that reliable quantification of cell wall components, especially the study of leaf C vs. Ca and Mg relationships, will prove valuable information for construction costs, leaf functions, and ecological strategies.

We measured leaf C, N, P, K, Ca, Mg concentrations and calculated leaf protein-free carbon concentration (*C*_S_) at the individual tree, species, and ecosystem scales. We hypothesized that there was a negative correlation between leaf C and Ca concentrations, and that this relationship reflects similar load-bearing roles of lignin and Ca^2+^-pectate with different carbon costs. We also hypothesized that the key leaf functional dimensions, structural and metabolic, can be characterized by leaf macronutrients. Specifically, that leaf carbon and calcium concentrations characterize the leaf structural axis, and nitrogen, phosphorus, and potassium concentrations characterize the leaf metabolic axis. Finally, we explored the C–Ca tradeoff in the cell wall by looking at leaf C and Ca correlations with individual cell wall components.

## Materials and Methods

### Research Sites

We investigated 15 broad-leaved forest sites across a wide range of environmental gradients in China (Supplementary Figure 1). We chose eight temperate deciduous broad-leaved forests (DBFs) sites at the Loess Plateau, including Mt. Guanshan, Mt. Taibaishan, Mt. Ziwuling, and Mt. Huanglong in Shaanxi Province, and Mt. Luyashan, Mt. Guandishan, Mt. Taiyueshan, and Mt. Taihangshan in Shanxi Province. The mean annual temperature (MAT) ranged from 4.1 to 10.3°C across these sites. At the karst hill, we chose three sites for subtropical mixed deciduous and evergreen broad-leaved forests (MDEBFs) at Puding County (MAT: 15.1°C), Guizhou Province. We investigated four sites from tropical-subtropical evergreen broad-leaved forests (EBFs) in Southern China, and MAT ranged from 20.7 to 23.6°C, including Mt. Dinghu, Mt. Wutong in Guangdong Province, Mt. Jianfeng in Hainan Province, and the Xishuangbanna rainforest in Yunnan Province.

### Leaf Sampling and Measurements of Elements and Cell Wall Components

We collected leaf samples during the growing season (mainly in July and August) from 2014 to 2016. We randomly sampled three healthy broad-leaved trees for each species inside the forest and collected 20 upper canopy sun-exposed leaves of each tree from the top part of the canopy. Leaf samples were oven dried at 80°C for 24 h, pulverized using a sample mill, and then sieved through a 0.15-mm mesh screen. We determined the leaf C and N concentrations (mg g^−1^) for each sample by a CHNS analyzer (Vario EL III; Elementary Analyze system GmbH, Hanau, Germany). The leaf samples were dissolved in 65% nitric acid (HNO_3_), and then we measured P, K, Ca, and Mg concentrations (mg g^−1^) by inductively coupled plasma atomic emission spectrometry (ICP-AES, Spectro, Germany).

To detect the cell wall constitution, we recollected the leaf samplings in our two typical MDEBF and EBF sites in August 2020. We chose the 20 most common angiosperm tree species from a similar subtropical climate but with different bedrocks (red sandy conglomerate vs. karst limestone). At each site, five evergreen species and five deciduous species were sampled with three individuals for each species, respectively. For hemicellulose, cellulose, and lignin measurement, 1.5-g fresh leaf laminas were cleaned with distilled water and homogenized in a 0.1-M sodium phosphate buffer (pH 7.5). The homogenate was centrifuged at 2,000 *g* for 10 min. The pellet containing cell-wall materials with a small amount of starch was kept. To remove starch, amyloglucosidase (35 units mL^−1^) was added and incubated at 55°C for 30 min (Rufty and Huber, 1983). The cell-wall material was washed four times with distilled water and twice with ethanol, each time followed by centrifugation (2,000 *g* for 10 min); finally, it was dried in an oven (60°C) for 48 h. After drying, hemicellulose, cellulose, and lignin concentrations in the cell wall were measured in a series of steps. After boiling in 30 ml of neutral detergent (1% w/w sodium dodecyl sulfate) for 1 h, neutral detergent fiber (NDF) was collected and washed four times with hot (90°C) distilled water. With 20 ml of 2-M HCl added and boiling in a water bath for 1 h, the filtrate of acid detergent fiber (ADF) was collected, pH was adjusted to 5 with NaOH, and hemicellulose concentration was determined by the 3,5-dinitrosalicylic acid colorimetric method (Jain et al., 2020). The residue was rinsed by pure water four times, hydrolyzed for 1 h in 20 ml of 72% w/w sulfuric acid, and then diluted to 100 ml. The filtrate was measured by 2% w/w anthrone-sulfuric acid colorimetry to determine cellulose concentration. The final residue was oven dried at 105°C, representing lignin in the cell wall. We measured Ca- and Mg-bounded pectins protopect into be exact concentrations by the standard method according to a dilution ratio (Dische, 1947; McFeeters and Armstrong, 1984). The Ca in cell walls was extracted by Tris-HCl, saccharose, DTE (C_4_H_10_O_2_S_2_), ascorbic acid, buffer solution, and centrifuged to get the residual cell wall fragments, and then measured Ca concentration in the residue (*Ca*_cellwall_) by inductively coupled plasma atomic emission spectrometry (ICP-AES, Spectro, Germany). The C concentration in the leaf cell wall (*C*_cellwall_) was calculated according to the proportions of lignin (62.5% C), hemi- and cellulose (44.4% C), and protopectin [(C_6_H_10_O_7_)_n_, 37% C] in the cell wall.

Because leaf proteins comprising most leaf N generally possess higher C concentration (53.5%) than the leaves [around 50% on average at the global scale (Ma et al., 2018)], we calculated protein-free C concentrations (*C*_S_) of the leaves, as described in Niinemets and Tamm (2005).

(1) $$\begin{array}{r} C_{\text{S}}=\frac{C-6.25\left(\frac{53.5N}{100}\right)}{1-\frac{6.25N}{1000}} \end{array}$$

*C* is the total leaf C concentration, *N* is the leaf N concentration, and 6.25 converts the leaf N concentration to protein concentration.

### Collection of Environment Variables

Based on the actual mean month temperature and the precipitation from CRU TS v. 4.03 dataset released on 15 May 2019, covering the period of 1999–2018, with spatial resolution of 0.5° × 0.5° (latitude × longitude) grid cells (Harris, 2019; Harris et al., 2020), we calculated MAT and MAP (mean annual precipitation) for all sampling sites. We extracted the available N, P, K, and exchangeable Mg^2+^, Ca^2+^, and soil pH data in 0–30-cm soil depth from “the China dataset of soil properties” (Shangguan and Dai, 2013), using ArcGIS 10.2.

### Data Analyses

We considered the data from the same species from the same site as one species by a site, following Han et al. (2005) and Wright et al. (2005). We collected a total of 335 species by sites (222 species) for C, N, P, K, Ca, and Mg concentrations of leaves averaged for 15 forest ecosystems. We collected 90 samples for *Quercus wutaishanica* and 47 samples for *Betula platyphylla* from different locations and measured leaf chemical traits to determine intraspecific variations. Cell wall chemical structures for 20 broad-leaved tree leaves were further measured.

Analysis of variance (ANOVA) was conducted with SPSS 23.0 (SPSS Inc., Chicago, IL) to compare the differences in leaf C, *C*_S_, N, P, K, Ca, and Mg among different species by a site [using one-way ANOVA with Games–Howell *post-hoc* tests], and among individual groups of the two species (using one-way ANOVA between groups). Pairwise correlation analysis was performed to explore correlations among the log_10_-transformed C, *C*_S_, and macronutrient concentrations in the leaves. We employed principal component analysis (PCA) to explore multiple-trait relationships, using CANOCO 5 (Microcomputer Power, Ithaca, NY, USA). The “biogeochemical niche” characterized the species position in the multivariate space generated by species nutrients concentrations (Peñuelas et al., 2008), and we applied this concept in this study based on two groups of PCAs. We conducted PCA for *C*_S_ and Ca and got PC1 scores of leaf *C*_S_ and Ca concentrations (PC1_C*sCa*_), and the other of P and K and got PC1 scores of leaf P and K concentrations (PC1_PK_); and then we tested correlations among the PC1_C*sCa*_, PC1_PK_, leaf N, and Mg concentrations by using SPSS 23.0 (SPSS Inc., Chicago, IL). Pearson's correlations and phylogenic independent contrast (PIC) were used to analyze the relations among C, Ca, lignin, and protopectin concentrations in the cell wall and between C and Ca concentration in a leaf.

## Results

### The Variation of Leaf Macronutrients Across Intra-, Interspecies, and Ecosystem

Subtropical evergreen broad-leaved forests (EBF) showed the highest leaf C and *C*_S_ concentrations and the lowest Ca, Mg, and N concentrations. In contrast, mixed deciduous and evergreen broad-leaved forests (MDEBF) from karst hills tended to have the highest leaf Ca and Mg but lowest C and *C*_S_ concentrations (Table 1). We detected a significant difference in leaf C, *C*_S_, P, Ca, and Mg concentrations between evergreen and deciduous tree species even within MDEBF (Table 1). Between individuals of two species, the higher Ca but lower C, *C*_S_, K, and Mg concentrations were significantly observed in the leaves of *Q. wutaishanica* than those of *B. platyphylla*, whereas the differences in leaf N and P concentrations were not significant (Table 1). Generally, leaf C and *C*_S_ concentrations had the lowest coefficient of variations (CVs) than leaf macronutrient concentrations (Table 1).

**Table 1:** Leaf carbon (C), protein-free carbon (C_S_), and macronutrient concentrations (mg g^−1^) for samples from different scales and plant functional types.

**Research groups**		**Sample size**	**C**			***C***_****S****_			**N**			**P**			**K**			**Ca**			**Mg**		
		**Mean**	**SE**	**CV**	**Mean**	**SE**	**CV**	**Mean**	**SE**	**CV**	**Mean**	**SE**	**CV**	**Mean**	**SE**	**CV**	**Mean**	**SE**	**CV**	**Mean**	**SE**	**CV**	
Ecosystems		15	467	2.97	2.5	456	3.58	3.0	21.6	0.77	14	1.16	0.07	23	11.0	0.36	13	14.5	1.16	31	2.89	0.12	16
Species-by-sites	MDEBF	113 (Total)	456^A^	1.94	4.5	444^A^	2.30	5.5	20.6^B^	0.60	31	1.15^B^	0.34	30	11.8	0.49	44	19.3^C^	0.68	37	3.49^C^	0.15	44
		60 (Deciduous)	448^a^	2.12	3.7	435^a^	2.48	4.4	21.9	0.78	28	1.23^b^	0.04	25	12.6	0.73	45	20.8^b^	0.81	30	3.80^b^	0.20	41
		53 (Evergreen)	464^b^	2.91	4.7	455^b^	3.53	5.7	19.2	0.88	33	1.05^a^	0.05	33	10.9	0.63	42	17.5^a^	1.08	45	3.11^a^	0.20	46
	DBF	137	465^B^	1.44	3.6	454^B^	1.65	4.3	23.3^C^	0.36	21	1.23^B^	0.04	35	11.0	0.31	33	15.5^B^	0.45	34	2.96^B^	0.07	27
	EBF	85	481^C^	3.00	5.8	474^C^	3.36	6.5	17.9^A^	0.79	41	1.00^A^	0.05	45	10.3	0.67	60	9.90^A^	0.59	55	2.37^A^	0.14	56
Within species	*Qw*	90	470^i^	0.78	1.6	459^i^	0.88	1.8	23.6	0.36	15	1.17	0.03	22	6.87^i^	0.15	21	11.2^ii^	0.33	27	2.34^i^	0.07	26
	*Bp*	47	498^ii^	1.43	2.0	492^ii^	1.70	2.4	25.1	0.52	14	1.14	0.04	24	9.52^ii^	0.40	29	8.71^i^	0.39	31	2.75^ii^	0.09	21

*Leaf stoichiometric traits, including Leaf C, C_S_, and macronutrient concentrations, of 15 forest ecosystems were displayed with average values of all species, standard errors, and coefficient of variation (CV, %). Leaf stoichiometric traits were also displayed for individuals of two widespread deciduous broad-leaved trees, species by sites of different leaf types from mixed evergreen and deciduous broad-leaved forests (MDEBF) species by sites from three different forest types. Qw, Quercus wutaishanica; Bp, Betula platyphylla; DBF, the deciduous broad-leaved forest; EBF, the evergreen broad-leaved forest. Each species-by-site sample is the average value of leaf stoichiometric data (mg g^−1^) from no <3 individuals of each species from the same site, and each species sample is the average value of leaf stoichiometric data from all individuals of each species, and individuals are from leaves of each adult trees. Differences in leaf C, C_S_, and macronutrient concentrations among species by site of different forest types as well as individuals within two species were tested with the one-way ANOVA analysis. Compared groups are mostly or strictly with the normal distribution. Besides, we choose the Games–Howell post-hoc test results because all the results of homogeneity of variances tests are P ≤ 0.05. Results were presented with the upper letter, lower letter, and lower Roman at P ≤ 0.05 level*.

### The Tradeoffs Between C and Ca From Intra-, Inter-Specific, and Ecosystem Scales

**Figure 1:**
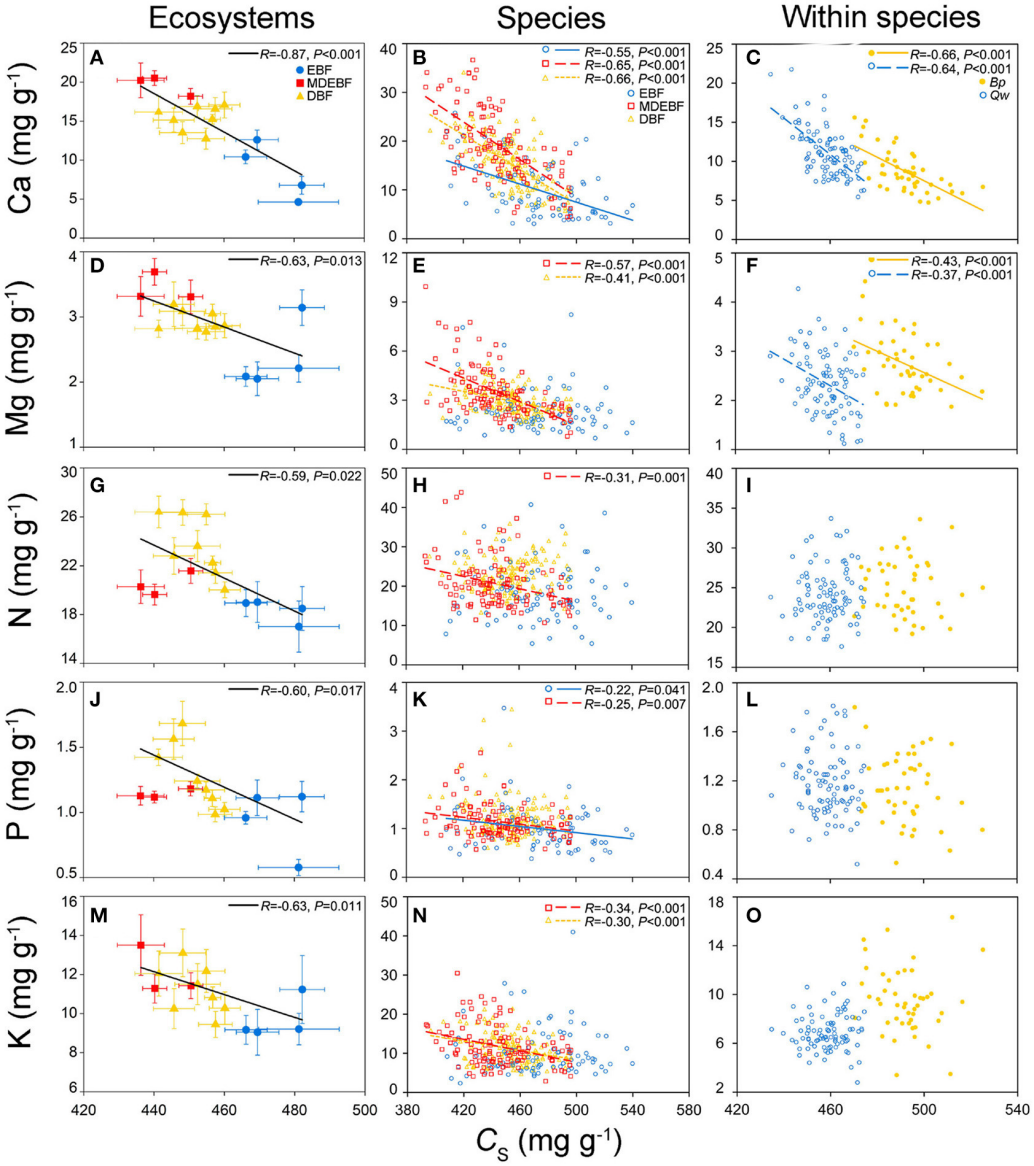
Relationships between leaf protein-free carbon (C_S_) and macronutrient concentrations at the ecosystem, species, and within species scales. Filled circles/squares/triangles represent average value with the standard division of leaf C_S_ and macronutrients from the evergreen broad-leaved forest (EBF), mixed deciduous and evergreen broad-leaved forests (MDEBF), deciduous broad-leaved forest (DBF), and continuous lines represent regressions between leaf C_S_ and macronutrients at ecosystem scale (*P* ≤ 0.05) **(A,D,G,J,M)**, error bars represent standard errors of the mean. Open circles/squares/triangles and continuous/dashed/dotted lines inspecies groups represent samples from EBF/MDEBF/DBF (*P* ≤ 0.05) **(B,E,H,K,N)**. Filled/open circles and continuous/dashed lines at within species scale represent samples of Quercus wutaishanica (Qw), Betula platyphylla (Bp) **(C,F,I,L,O)**. R is Pearson's correlation coefficient.

Leaf Ca was the only trait that was consistently negatively related with both leaf C and *C*_S_ (Figure 1, Supplementary Tables 1, 2). Leaf Mg concentration of MDEBF, DBF, and within two species was positively correlated with leaf Ca concentration; however, for EBFs, leaf Mg was correlated with leaf P and K concentrations more tightly (Figure 1, Supplementary Tables 1, 2) than that with leaf Ca. Leaf N of different groups did not demonstrate a consistent relationship with other leaf chemical traits (Supplementary Tables 1, 2).

### The Relations Between Leaf Macronutrients and Environmental Drivers

**Figure 2:**
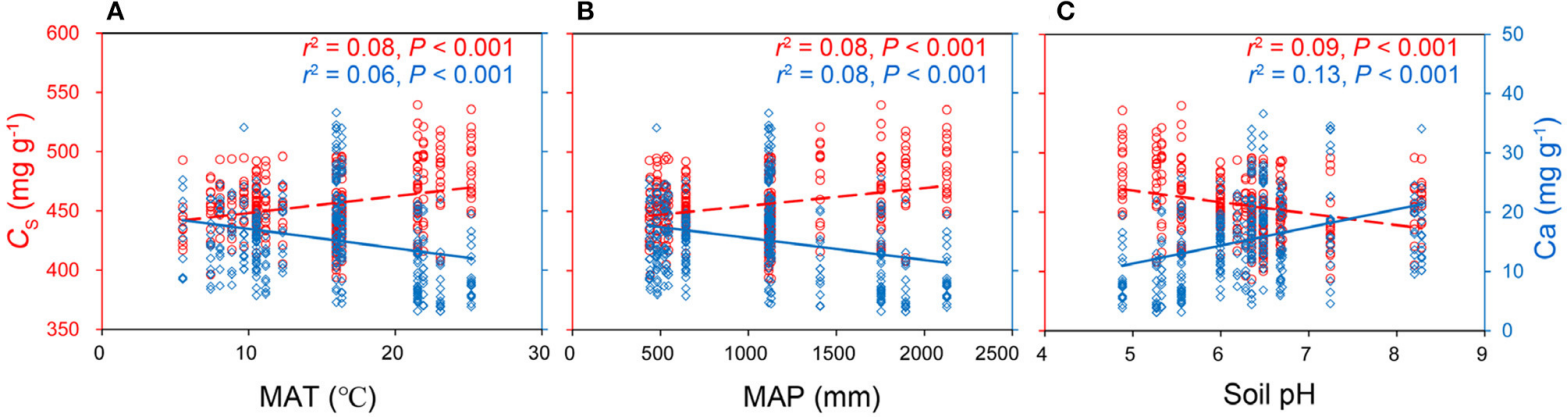
The double y-axis graphs explaining the relationships between leaf protein-free carbon (C_S_), calcium (Ca) concentrations (mg g^−1^), and mean annual temperature (MAT) **(A)**, mean annual precipitation (MAP) **(B)**, and soil pH **(C)** at species scale (*n*: 335 species by sites). The red left y-axis indicated leaf C_S_, the blue right y-axis indicated leaf Ca. Equations of linear relationships: C_S_ = 1.42MAT + 434 (*r*^2^ = 0.08, *P* < 0.001); Ca = −0.33MAT + 20.2 (*r*^2^ = 0.06, *P* < 0.001);C_S_ = 0.015MAP + 440 (*r*^2^ = 0.08, *P* < 0.001); Ca = −3.69 × 10^−3^ MAP + 19.2 (*r*^2^ = 0.08, *P* < 0.001);C_S_ = −9.6pH + 516 (*r*^2^ = 0.09, *P* < 0.001); Ca = 3.06pH – 3.98 (*r*^2^ = 0.13, *P* < 0.001).

MAT, MAP, and soil pH were the critical climate and soil drivers for the variation of leaf *C*_S_ and Ca (Figure 2, Supplementary Table 3). Both leaf C and *C*_S_ increased with MAT and MAP but decreased with soil pH across 335 species by sites, and leaf Ca showed the opposite trends along with these environmental factors (Figure 2). Both leaf C and *C*_S_ significantly increased with the MAT increasing. Leaf N was significantly affected by soil nutrients instead of climate variables within species (Supplementary Table 3). Interestingly, soil pH explained more leaf C, *C*_S_, and Ca variations than MAT (Figure 2, Supplementary Table 3). Environmental factors, such as MAT, MAP, soil available N, P, K, Mg, and soil pH, explained no more than 10% variations for leaf C, *C*_S_, P, K, Mg (*R*^2^ < 0.10) at a species scale.

### Decoupling Between Leaf C–Ca and Leaf P–K Concentrations

**Figure 3:**
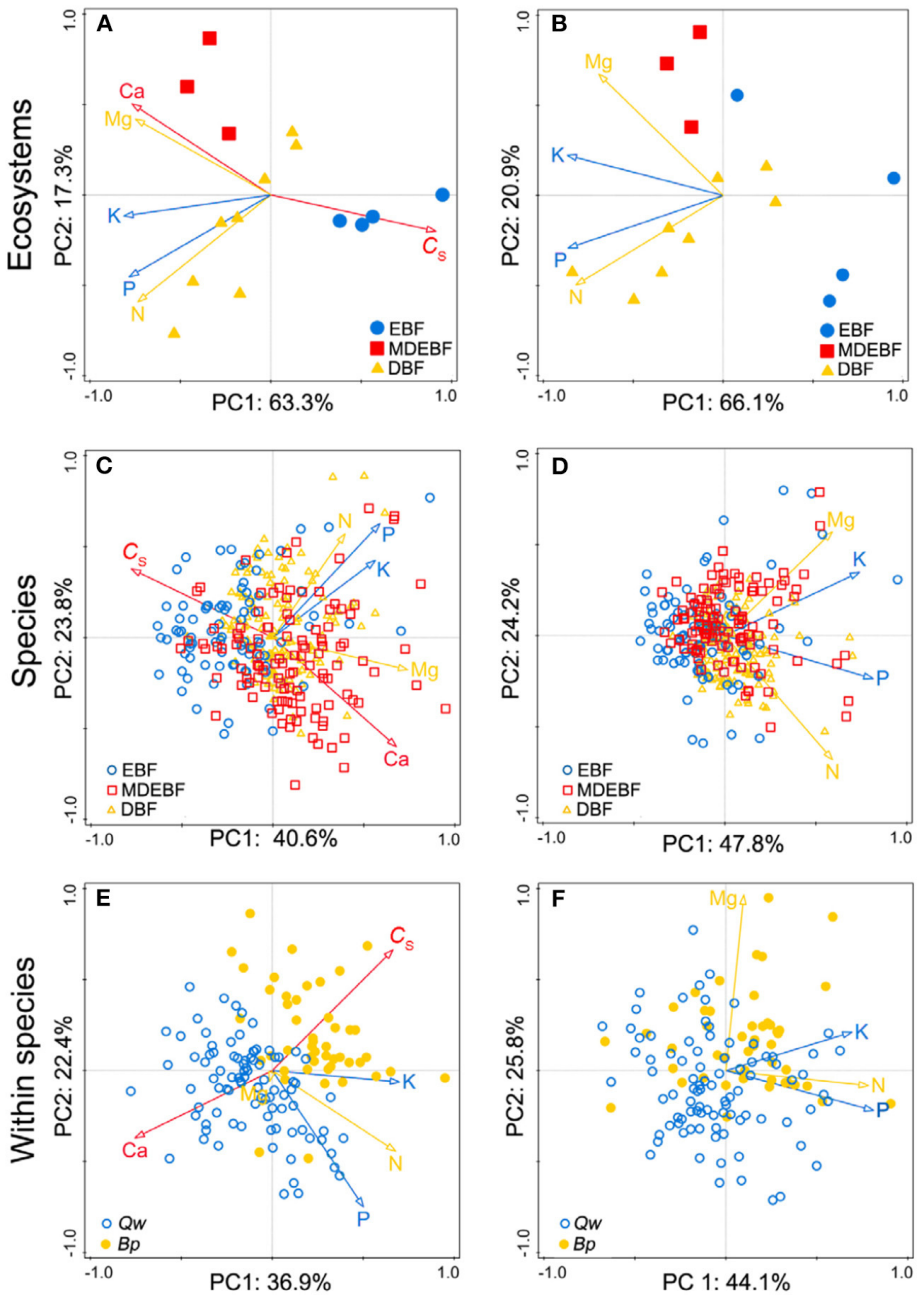
Comparisons between leaf macronutrients with leaf protein-free carbon (C_S_)-calcium (Ca) **(A,C,E)** and macronutrients without C_S_-Ca **(B,D,F)** across six principal component analyses. The red arrows suggested cell wall-related leaf traits (C_S_ and Ca concentrations), blue arrows showed the protoplasm-related leaf traits [phosphorus (P) and potassium (K) concentrations], and yellow arrows were nitrogen (N) and magnesium (Mg) concentrations. Samples of species by site from EBF/MDEBF/DBF were displayed by open circles/squares/triangles in green/blue/yellow colors **(C,D)**. Individuals of Quercus wutaishanica (Qw), Betula platyphylla (Bp) were displayed by open/filled circles in green/yellow colors **(E,F)**. Abbreviations of sample groups are described in Figure 1. Numeric values in axis labels show the percentage of total variance explained by each axis.

**Figure 4:**
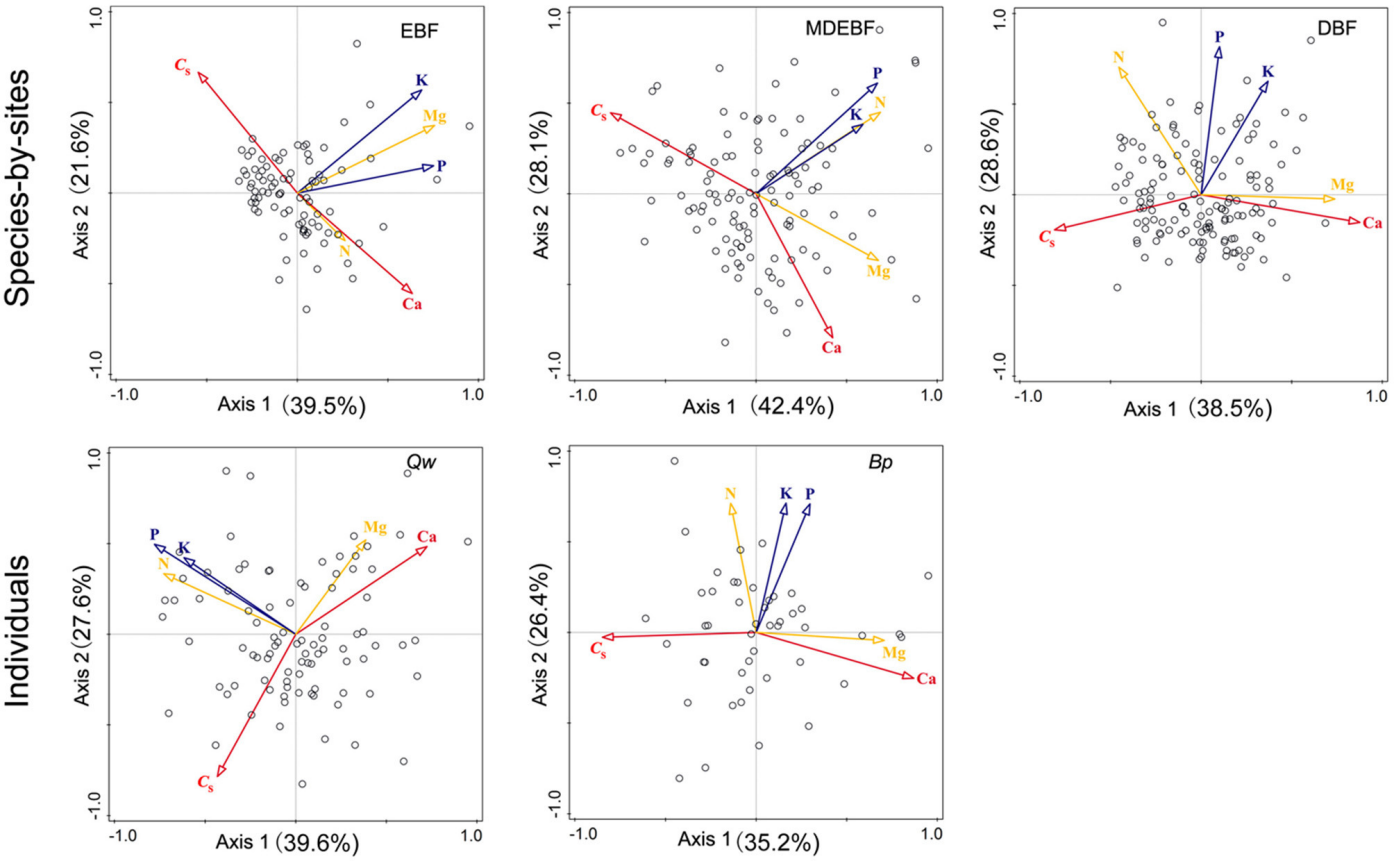
Principal component analyses (PCAs) of leaf protein-free carbon (C_S_) and macronutrient concentrations for individuals of Quercus wutaishanica, Betula platyphylla, and species by sites from EBF, MDEBF, and DBF. Abbreviations of sample groups and explanation of trait arrows are described in Figure 1.

We observed biogeochemical niche differentiation across intra-, interspecies, and ecosystems based on two grouped PCAs (Figure 3). One grouped PCA was conducted with leaf *C*_S_, N, P, K, Ca, and Mg (Figures 3A,C,E); the other grouped PCA was conducted with leaf N, P, K, Mg without *C*_S_ and Ca (Figures 3B,D,F). In this way, we distinguished the cell wall-related traits (including leaf *C*_S_ and Ca) and protoplasm-related traits (including leaf P and K) (Figure 4). Based on the PCAs across intra-, interspecies, and ecosystems, the first and second axes accounted for 61–71% total variation of leaf *C*_S_, N, P, K, Ca, and Mg concentrations (Figure 4, Table 2). We did not find significant correlations between PC1_C*sCa*_ and PC1_PK_ (*P* > 0.05, Supplementary Table 4), indicating decoupled between leaf cell wall elements and cell wall protoplasm elements. We found strong correlations between leaf Ca and Mg, except for EBF (Figure 4, Supplementary Table 1). Leaf Mg was tightly correlated with PC1_C*sCa*_ for most research groups except that of EBF species by sites; analogously, the leaf N did not show a consistent correlation with PC1_C*sCa*_, or PC1_PK_ from different groups (Supplementary Table 5).

**Table 2:** Extraction sums of squared loadings and loading scores of leaf C_S_, Ca, K, P, and N concentrations in the PCAs for individuals of two species and species by sites of three forest types.

		**% of explained variance**		***C*_**S**_**	**N**	**P**	**K**	**Ca**	**Mg**
Species-by-sites	EBF	Axis 1	39.5	**−0.55**	0.23	**0.75**	**0.69**	**0.63**	**0.76**
		Axis 2	21.6	**0.67**	−0.24	0.15	**0.57**	**−0.56**	0.36
	MDEBF	Axis 1	42.4	**−0.80**	**0.69**	**0.67**	**0.59**	0.42	**0.67**
		Axis 2	28.1	0.45	0.45	**0.61**	0.38	**−0.79**	−0.37
	DBF	Axis 1	38.5	**−0.81**	−0.48	0.04	0.37	**0.86**	**0.74**
		Axis 2	28.6	−0.20	0.32	**0.87**	**0.70**	−0.21	−0.04
Individuals	*Qw*	Axis 1	39.6	−0.43	**−0.73**	**−0.78**	**−0.62**	**0.72**	0.39
		Axis 2	27.6	**−0.78**	0.33	0.50	0.42	0.48	0.52
	*Bp*	Axis 1	35.2	**−0.85**	−0.14	0.30	0.17	**0.87**	**0.71**
		Axis 2	26.4	−0.03	**0.71**	**0.71**	**0.71**	−0.25	−0.04

*EBF, broad evergreen forest; DBF, deciduous broad-leaved forest; MDEBF, mixed deciduous and evergreen broad-leaved forest; Qw, Quercus wutaishanica; Bp, Betula platyphylla. Bold numbers show the major loading of leaf traits on axes*.

### Relationships Among C_cellwall_, Ca_cellwall_, and Lignin, Protopectin Concentrations in Cell Wall

**Figure 5:**
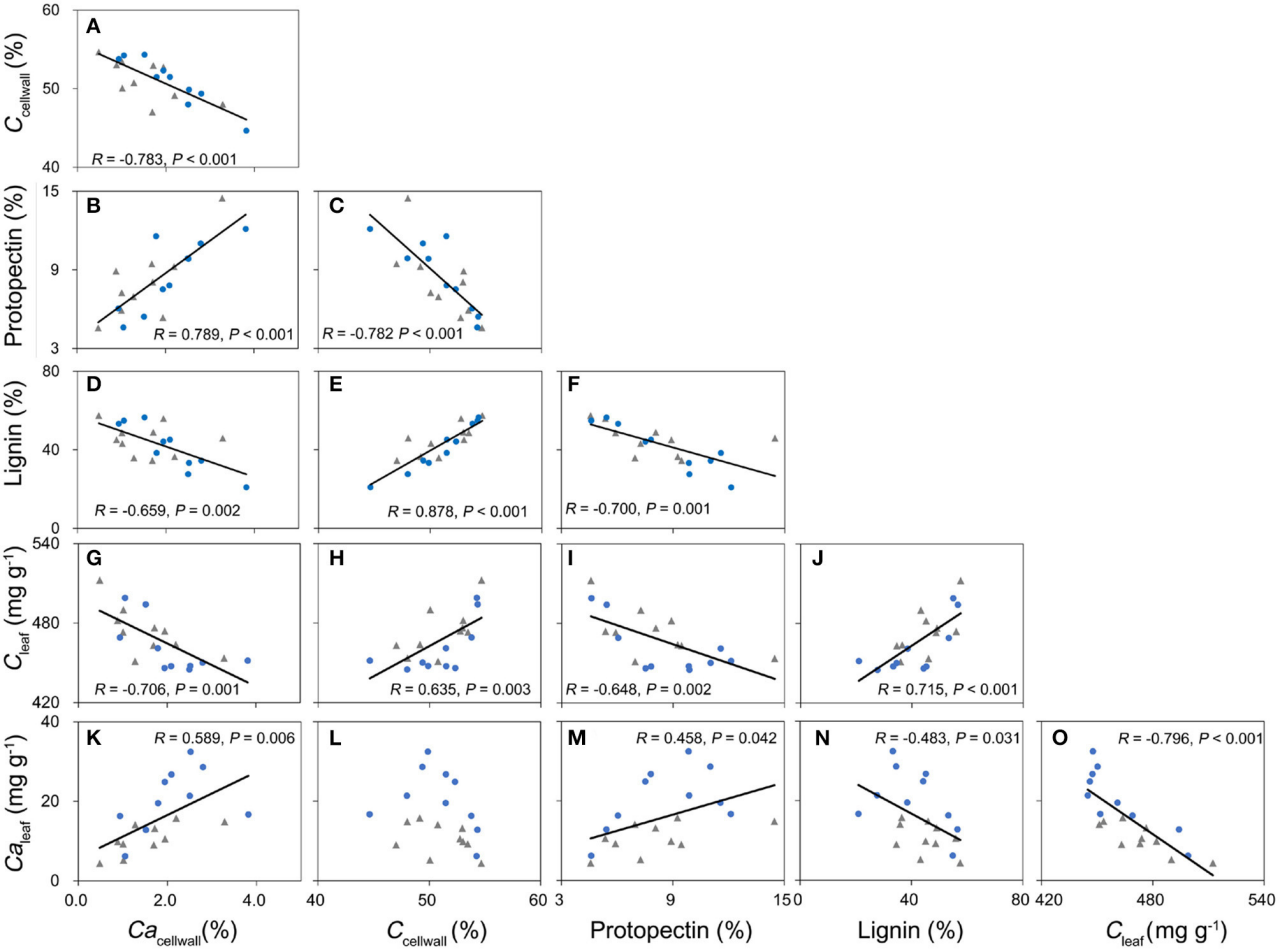
The relationships among carbon in the leaf cell wall (C_cellwall_), calcium in the leaf cell wall (Ca_cellwall_), lignin, and protopectin in the leaf cell wall, leaf carbon concentration (C_leaf_), and leaf calcium concentration (Ca_leaf_) of 20 broad-leaved tree species **(A–O)**. R: Pearson's correlation coefficients. Filled blue circles are ten species from the non-karst sampling site, and filled gray rhombs are ten species from the karst sampling site. Solid lines correspond to significant correlations for paired traits (*P* ≤ 0.05).

*Ca*_cellwall_ (Ca concentration in the cell wall) was negatively related with *C*_cellwall_ based on Pearson's correlation and phylogenic independent contrast (PIC) (Figure 5A, Supplementary Table 6) across 20 broad-leaved woody dicots. In contrast, *Ca*_cellwall_ was positively correlated with protopectin concentration and negatively correlated with lignin concentration (Figures 5B,E, Supplementary Table 6), but lignin concentration and protopectin concentration in cell walls were negatively correlated (Figure 5F).

## Discussion

We found consistently negative correlations between leaf *C*_S_ (and C) and Ca concentrations across inter-, intra-specific, and ecosystem levels. The leaf C–Ca tradeoff reflected the optimal cell wall compositions and structural cost-efficiency in leaf construction, and suggested shifts in investment strategies originated from a tradeoff between lignin and protopectin in the cell wall. The decoupled pattern between structure- and metabolism-related macronutrients would be explained by the fundamental principle of the leaf design of the cell wall and cell protoplasm. Our results suggested leaf structural strategies resulting from optimization of C–Ca interactions in response to climate and soil conditions.

In our study, leaf C–Ca tradeoffs were observed at different scales of biological organization from cells to ecosystems. Previously, such a relationship has been observed for the temperate broad-leaved deciduous tree *Populus tremula* (Niinemets and Tamm, 2005). We also demonstrated that this tradeoff represents different leaf structural strategies as driven by the share of lignin and protopectin in the construction of the cell wall (Figure 5A). We also observed that lignin concentration was positively related to leaf C concentration (Figures 5H,J), indicating that it is expensive to invest more C to build mechanically resistant cell walls with more lignin. High C cost is less limiting under improved resource supply, e.g., in conditions of the extended growing season and water availability that both enhance leaf C content (Ma et al., 2018). Indeed, in our study, leaf C was positively related to MAT and MAP across 335 species by sites (Figure 2, Supplementary Table 3).

**Figure 6:**
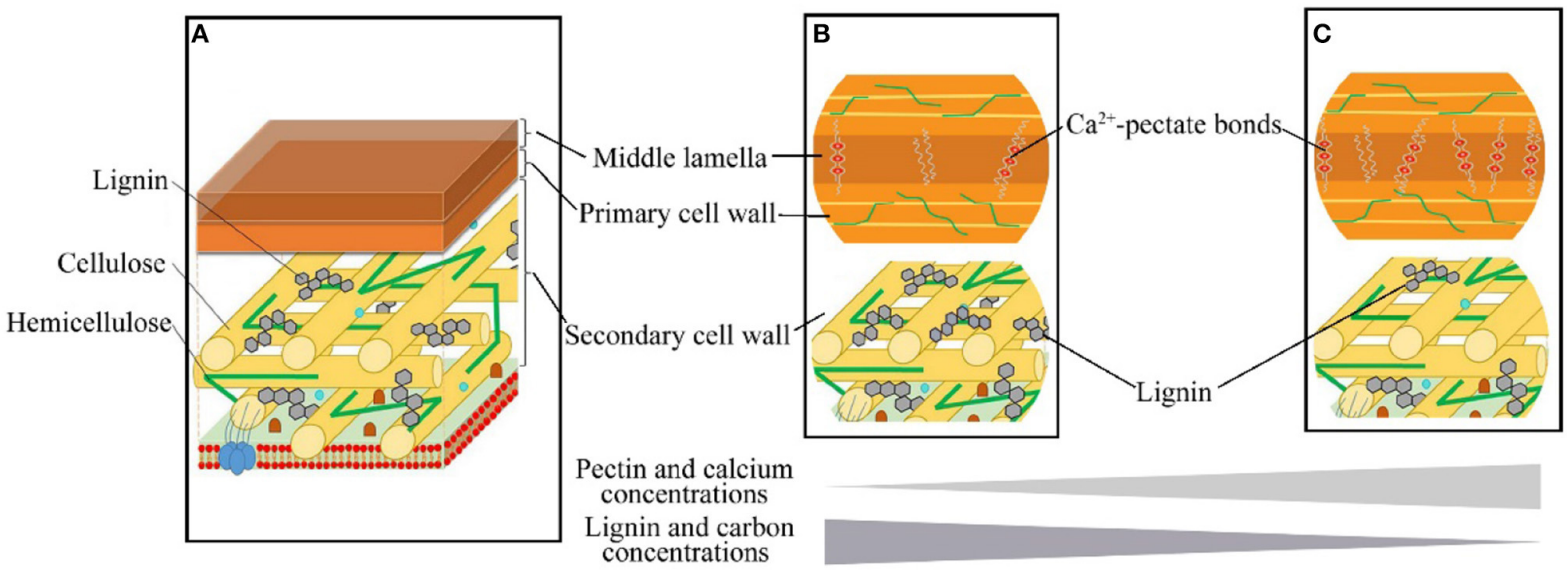
The leaf cell wall composition and structural tradeoffs of woody dicots. **(A)** The middle lamella, mainly made up of Ca^2+^-pectate, is the outermost layer of the cell wall; the primary cell wall, mainly consist of cellulose and hemicellulose, is located outside of the plasma membrane; the secondary cell wall is located between the primary cell wall and the plasma membrane, mainly consist of lignin, cellulose microfibrils [referencing to Loix et al. (2017)]. **(B,C)** indicated the tradeoff between Ca and C in structural strategies. **(B)** Cell wall build by less Ca^2+^-pectate in middle lamella but more lignin **(C)** in the secondary cell wall. (and C) Cell wall build by more Ca^2+^-pectate in middle lamella but less lignin (and C) in the secondary cell wall.

Although higher MAT and MAP generally increase a plant growth rate and leaf C concentration at the same time, higher MAT and MAP enhance the activity of herbivores (Onoda et al., 2011; Chen et al., 2019). Therefore, leaves with ample C tend to invest more in lignin to build cell walls that are more resistant to herbivory, allowing to extend a leaf lifespan (Villar et al., 2006; Popper, 2011). In contrast, Ca^2+^-pectate is the cheapest structural component for cell wall construction (Poorter et al., 1997), but these cheaper leaves with higher Ca are typically more palatable for herbivores (Mládková et al., 2018). Thus, the low-cost leaves with lower C but higher Ca concentrations can grow and mature fast, and be more competitive in climates with a short growing season or in highly unpredictable climates, especially when the soil Ca supply is sufficient. For example, with enough Ca supply from karst hills, most calciphilic plants built leaves with more Ca but less C (Figure 6, Table 1). Thus, leaf structural strategies could be optimized by soil Ca supply. The increase of leaf Ca with increasing soil pH (Figure 2C) is consistent with previous studies (Tyler and Olsson, 2001; Han et al., 2011). In general, as soil pH decreases, Ca is released from Ca-rich minerals (such as calcite and dolomite) and Ca^2+^ is leached out of the soil profile (Tyler and Olsson, 2001). Therefore, soil pH determines the soil Ca supply and strongly influences the leaf *C*_S_-Ca tradeoff (Figure 2C). Overall, our study indicated that both leaf chemical constitution and cell wall structure were regulated by MAP, MAT, and soil Ca supply (Figures 2, 3, Supplementary Table 3). Furthermore, leaf structural strategies (investment in lignin vs. investment in protopectin) were fundamentally linked with the generally overlooked C–Ca tradeoff.

Our study further proposed that leaf structural and metabolic strategies can be captured by leaf stoichiometric dimension space. We identified decoupled dimensions of leaf cell-protoplasm metabolic axis (represented by P and K concentration) and cell-wall structural axis (including *C*_S_ and Ca concentrations) from species to forest ecosystem (Figure 4). Two independent axes described 60–80% of the variation within and across species and ecosystems (Table 2). High loading of leaf C and Ca concentrations on axis 1 revealed a tradeoff between leaf C and Ca structural investments, consistent with their contributions to the cell wall structure. This leaf structural dimension (axis 1) stands for leaf load bearing, and physical defense functions, which were primarily linked with the constitution of lignin, (hemi)cellulose, and Ca^2+^-pectate in the cell wall, which will be predicted by leaf C concentrations. The leaf metabolic dimension (axis 2) revealed covariation among P, K, and Mg for cell metabolic and leaf physiological investment, which we term the leaf cell protoplasm metabolic axis. The metabolic process is inherently linked with cell protoplast, which is termed as a naked cell, excluding the cell wall and constituted by the living nucleus, cytoplasm, plastids, and mitochondria of the cell (Raven et al., 2005). Thus, P and K concentrations indicated the metabolic status of the cell protoplast. Overall, C–Ca structural axis and metabolic P-K axis traits varied independently (Figure 4, Supplementary Table 4), giving a leaf much more freedom to adjust its functioning to adapt to local resource-rich patches. In this way, diverse leaf construction strategies during evolution might promote species coexistence in a forest community according to heterogeneous resource supplies.

We did not detect consistent patterns of leaf C–N and C–Mg from cell to ecosystems. N and Mg formed both structural and metabolic components; these two elements' concentrations were difficult to indicate the structural function or metabolic intensity (Figure 4, Supplementary Table 5). However, the Mg concentrations were well-correlated with leaf *C*_S_ concentrations in samples of most groups, except those from the evergreen broad-leaved forest (Figures 2, 3). Structural Mg is bonded to pectate (Mg^2+^-pectate) in the cell wall (5–10%), and the rest of the Mg exists as Mg^2+^ and sparingly soluble salts in the cell cytoplasm (Hawkesford et al., 2012). The primary function of Mg in the protoplasm of green leaves is as the central atom of the chlorophyll molecule, and its proportion ranges from 6% in plants with a high Mg supply to 35% in Mg-deficient plants (Hawkesford et al., 2012). Some Mg exists as Mg^2+^ and sparingly soluble salts in the cell cytoplasm (Hawkesford et al., 2012). The tradeoffs between structural Mg^2+^-pectate and metabolic Mg-chlorophyll depend mainly on Mg nutritional status. For example, the percentage of Mg bound to chlorophyll increases with soil Mg deficiency (Rios et al., 2012). In Mg-deficient soil, the amount of Mg in chlorophyll can be up to 35% (Hawkesford et al., 2012) and even >50% in an Mg-deficient poplar (Dorenstouter et al., 1985; Hawkesford et al., 2012) and beech (*Fagus sylvatica*) (Niinemets, 1995). In our study, the lowest leaf Mg concentrations were observed in species from the EBFs (Table 1), and these moderately low concentrations suggested some degree of Mg deficiency in leaves. For most groups, except EBFs, leaf Mg was significantly correlated with the PC1_C*sCa*_ instead of the PC1_PK_ (Supplementary Table 5). These results suggested that leaf Mg variations were mostly caused by how much Mg^2+^-pectate was invested in the cell wall.

The variation of leaf N resulted from complex N-components combinations and heterogeneous distributed in both cell wall and protoplasm. The relationships between leaf N and other leaf element concentrations (Figure 1, Supplementary Tables 1, 2). PC1_C*sCa*_ and PC1_PK_ (Supplementary Table 5) were not consistent, which suggested N distribution in cells was substantial plastic. Fundamentally, N was responsible for multiple functions with multiple structures, and its biochemistry in the cell was complex (Hawkesford et al., 2012). Additionally, leaf N was inherently linked with plant functional types, such as N fixing vs. non-N fixing species. Leaf N variation was caused by its physiological and structural tradeoffs and driven by soil N availability, irradiation, and temperature (Reich and Oleksyn, 2004; Richardson, 2004; Onoda et al., 2017).

Leaf *C*_cellwall_ and Ca_cellwall_ were correlated to lignin, protopectin, hemicellulose, and cellulose concentrations at the cell scale. We provided structural and functional bases for leaf C variations at the cell level (Figure 6). Therefore, it is inappropriate to treat leaf C concentration as a relatively stable trait in stoichiometric studies. Leaf C concentration as an indicator of the tradeoff among different structural components in cell walls has been suggested as an essential trait in explaining ecological niche differentiation (Peñuelas et al., 2008, 2019). However, as our study indicates, leaf C together with Ca is a more informative indicator of leaf investment strategies than leaf C alone. To better understand leaf form diversity and species coexistence in ecological models, we should consider leaf structural and metabolic dimensions in leaf stoichiometry from cells to ecosystems. The decoupled multiple trait dimensions offer extensive freedom to enhance the efficiency of leaf constructions. Meanwhile, leaf traits flexibly provide alternate elements using strategies to adapt heterogeneous soil resource supplies and ensure species coexistence with full utilization of limited resources.

## Conclusions

We established a new leaf structural axis based on macronutrient stoichiometry. The structural macronutrient axis is decoupled from the leaf metabolic macronutrient axis and is characterized by consistent negative relationships between leaf *C*_S_ (and C) and Ca concentrations at intraspecific, interspecific, and ecosystem scales. The different combinations of structural C-components and Ca in the cell wall explain the negative leaf C–Ca relationships. Leaves with high Ca concentration contained much more protopectin and less C-rich lignin in the cell wall; such protopectin-rich leaves had lower leaf construction cost than lignin-rich leaves. Furthermore, biogeochemical niches differentiated species and ecosystems in response to soil Ca supply relating to soil pH, MAT, and MAP. Differently from C vs. Ca relationships, the relationships between C and N, and between C and Mg were inconsistent. This reflects differences in N and Mg distributions among cell wall and protoplasm as driven by nutrient supply. Re-exploring leaf nutrient stoichiometry, from cell to ecosystem scales by separately looking at leaf structural and metabolic dimensions, will lead to a better understanding of plant biogeochemical niches.

## Data Availability Statement

The raw data supporting the conclusions of this article will be made available by the authors, without undue reservation.

## Author Contributions

KX: conceptualization, methodology, sample collection, data curation and analysis, visualization, and writing the original draft. MZ: sample collection, data analysis, and writing—editing. ÜN: writing the original draft, reviewing, and editing. SN, HC, and PW: writing, reviewing, and editing. JT: writing—review and editing. DG: conceptualization and methodology. ZM: verification, supervision, writing, reviewing, editing, project administration, and funding acquisition. All authors contributed to the article and approved the submitted version.

## Conflict of Interest

The authors declare that the research was conducted in the absence of any commercial or financial relationships that could be construed as a potential conflict of interest.
