# Supplementary material for: Relationships Between Leaf Carbon and Macronutrients Across Woody Species and Forest Ecosystems Highlight How Carbon Is Allocated to Leaf Structural Function
Source: Front Plant Sci. 2021 Jun 11;12:674932. doi: 10.3389/fpls.2021.674932 (PMC8226226; doi:10.3389/fpls.2021.674932)
Supplement: Supplementary file 1 [file Data_Sheet_1.docx]

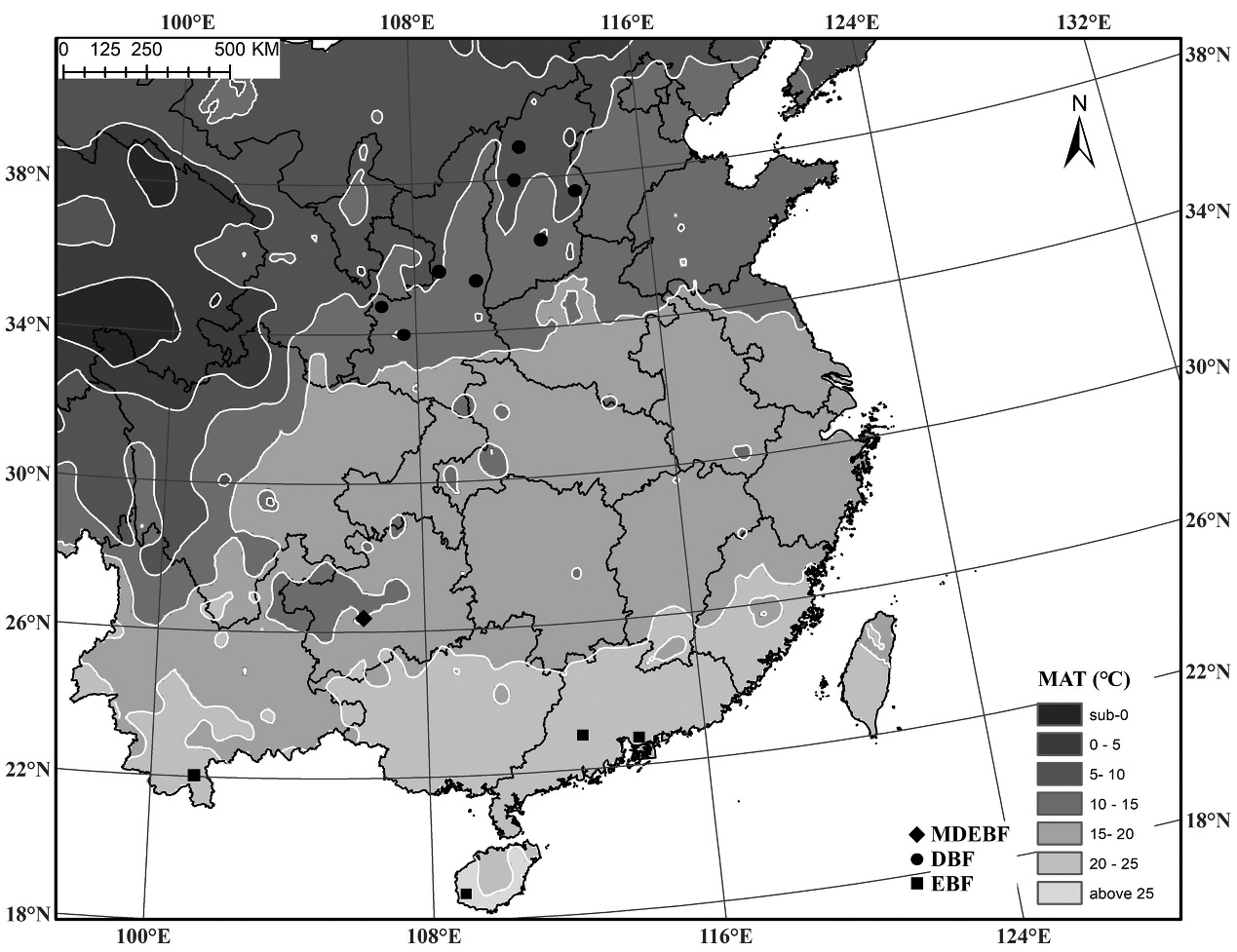
**FIGURE S1∣** Locations of the sampling sites in this study. The background is a portion of the Chinese mean annual temperature (MAT) map based on the meteorological data from 2395 weather stations over the last 30 years (from China meteorological data service centre, http://data.cma.cn/site/index.html). MDEBF, mixed deciduous and evergreen broadleaved forests; DBF, deciduous broadleaved forest; EBF, evergreen broadleaved forest.

**TABLE S1∣** Pearson's correlation coefficients among the leaf carbon (C), protein-free C (*C*_S_), nitrogen (N), phosphorus (P), potassium (K), calcium (Ca) and magnesium (Mg) concentrations of forests on average (top right part of total, *n* = 15) and species-by-site scale (bottom left part of total and different forest types).

|  |  | C | *C*_S_ | N | P | K | Ca | Mg |
| --- | --- | --- | --- | --- | --- | --- | --- | --- |
| Total | C |  | 1.00^***^ | -0.50 | -0.56^*^ | -0.60^*^ | -0.90^***^ | -0.64^*^ |
|  | *C*_S_ | 0.99^***^ |  | -0.59^*^ | -0.60^*^ | -0.63^*^ | -0.87^***^ | -0.63^*^ |
|  | N | -0.04 | -0.17^**^ |  | 0.76^**^ | 0.60^*^ | 0.33 | 0.25 |
|  | P | -0.19^**^ | -0.24^***^ | 0.43^***^ |  | 0.57^*^ | 0.38 | 0.44 |
|  | K | -0.17^**^ | -0.19^***^ | 0.16^**^ | 0.46^**^ |  | 0.43 | 0.64^*^ |
|  | Ca | -0.69^***^ | -0.69^**^ | 0.01 | 0.06 | 0.10 |  | 0.62^*^ |
|  | Mg | -0.43^***^ | -0.45^**^ | 0.12^*^ | 0.25^***^ | 0.35^***^ | 0.47^***^ |  |
| DBF | *C*_S_ | 0.99^***^ |  |  |  |  |  |  |
|  | N | 0.24^**^ | 0.12 |  |  |  |  |  |
|  | P | -0.10 | -0.15 | 0.38^***^ |  |  |  |  |
|  | K | -0.29^**^ | -0.30^***^ | 0.08 | 0.46^**^ |  |  |  |
|  | Ca | -0.70^***^ | -0.66^***^ | -0.43^***^ | -0.13 | 0.11 |  |  |
|  | Mg | -0.45^***^ | -0.41^***^ | -0.28^**^ | 0.08 | 0.22^**^ | 0.48^***^ |  |
| EBF | *C*_S_ | 0.99^**^ |  |  |  |  |  |  |
|  | N | 0.07 | -0.02 |  |  |  |  |  |
|  | P | -0.20 | -0.22^*^ | 0.18 |  |  |  |  |
|  | K | 0.10 | 0.09 | 0.02 | 0.47^***^ |  |  |  |
|  | Ca | -0.52^***^ | -0.55^***^ | 0.07 | 0.34^**^ | 0.14 |  |  |
|  | Mg | -0.10 | -0.11 | 0.07 | 0.44^***^ | 0.61^***^ | 0.26^*^ |  |
| MDEBF | *C*_S_ | 0.98^***^ |  |  |  |  |  |  |
|  | N | -0.13 | -0.31^**^ |  |  |  |  |  |
|  | P | -0.13 | -0.25^**^ | 0.69^***^ |  |  |  |  |
|  | K | -0.70^***^ | -0.34^***^ | 0.31^**^ | 0.48^***^ |  |  |  |
|  | Ca | -0.68^***^ | -0.65^***^ | <0.01 | -0.14 | -0.06 |  |  |
|  | Mg | -0.54^***^ | -0.57^***^ | 0.25^**^ | 0.20^*^ | 0.19^*^ | 0.39^***^ |  |

Abbreviations and sample sizes for each forest types are described in table 1. The statistical significance is indicated as: ^***^ ***P*** < 0.001; ^**^ 0.001 < ***P*** < 0.01; ^*^ 0.01 < ***P*** < 0.05.

**TABLE S2∣** Pearson's correlation coefficients between the leaf C, *C*_S_, N, P, K, Ca, and Mg concentrations for two deciduous broad-leaved tree species, including *Quercus wutaishanica* of 90 individuals and *Betula platyphylla* of 47 individuals.

|  |  | C | *C*_S_ | N | P | K | Ca |
| --- | --- | --- | --- | --- | --- | --- | --- |
| *Q. wutaishanica* | *C*_S_ | 0.98^***^ |  |  |  |  |  |
|  | N | 0.27^*^ | 0.05 |  |  |  |  |
|  | P | 0.07 | -0.07 | 0.64^***^ |  |  |  |
|  | K | 0.08 | 0.02 | 0.32^**^ | 0.61^***^ |  |  |
|  | Ca | -0.70^***^ | -0.64^***^ | -0.38^***^ | 0.32^**^ | -0.13 |  |
|  | Mg | -0.37^***^ | -0.37^***^ | -0.02 | 0.10 | -0.10 | 0.30^**^ |
| *B. platyphylla* | *C*_S_ | 0.99^***^ |  |  |  |  |  |
|  | N | 0.09 | -0.002 |  |  |  |  |
|  | P | -0.17 | -0.18 | 0.24 |  |  |  |
|  | K | -0.08 | -0.09 | 0.23 | 0.37^*^ |  |  |
|  | Ca | -0.69^***^ | -0.66^**^ | -0.34^*^ | 0.09 | 0.05 |  |
|  | Mg | -0.42^**^ | -0.43^**^ | 0.04 | 0.11 | -0.03 | 0.47^***^ |

Abbreviations and sample sizes for each forest types are described in table 1. The statistical significance is indicated as: ^***^ ***P*** < 0.001; ^**^ 0.001 < ***P*** < 0.01; ^*^ 0.01 < ***P*** < 0.05.

**TABLE S3∣** Ordinary least squares (OLS) regression lines fit the effects of environmental variables on leaf C, *C*_S_, nutrient concentrations at the ecosystem, species-by-site, and within species scales.

|  |  | Ecosystem | | | |  | Species-by-site | | | |  | Within species | | | |
| --- | --- | --- | --- | --- | --- | --- | --- | --- | --- | --- | --- | --- | --- | --- | --- |
|  |  | *R*^2^ | slope | intercept | *P* |  | *R*^2^ | slope | intercept | *P* |  | *R*^2^ | slope | intercept | *P* |
| Leaf C | MAT | 0.33 | 1.12 | 451 | 0.015 |  | 0.06 | 1.06 | 450 | <0.001 |  |  |  |  | 0.745 |
|  | logMAP |  |  |  | 0.063 |  | 0.04 | 19.6 | 454 | <0.001 |  | 0.11 | -148 | 881 | <0.001 |
|  | AN |  |  |  | 0.234 |  |  |  |  | 0.203 |  |  |  |  | 0.681 |
|  | AP |  |  |  | 0.229 |  |  |  |  | 0.056 |  |  |  |  | 0.222 |
|  | AK |  |  |  | 0.114 |  | 0.01 | -0.059 | 473 | 0.042 |  | 0.08 | 0.073 | 469 | 0.001 |
|  | Ca^2+^ | 0.29 | -1.53 | 475 | 0.023 |  | 0.03 | -0.96 | 471 | 0.002 |  |  |  |  | 0.230 |
|  | Mg^2+^ |  |  |  | 0.074 |  | 0.02 | -4.24 | 471 | 0.004 |  |  |  |  | 0.378 |
|  | pH | 0.27 | -6.64 | 510 | 0.028 |  | 0.08 | -7.81 | 515 | <0.001 |  |  |  |  | 0.679 |
| Leaf *C*_S_ | MAT | 0.40 | 1.46 | 435 | 0.007 |  | 0.08 | 1.42 | 434 | <0.001 |  |  |  |  | 0.727 |
|  | logMAP | 0.25 | 30.1 | 368 | 0.035 |  | 0.05 | 27.4 | 374 | <0.001 |  | 0.12 | -178 | 954 | 0.001 |
|  | AN |  |  |  | 0.196 |  |  |  |  | 0.114 |  |  |  |  | 0.473 |
|  | AP |  |  |  | 0.260 |  |  |  |  | 0.067 |  |  |  |  | 0.096 |
|  | AK |  |  |  | 0.081 |  | 0.02 | -0.087 | 466 | 0.01 |  | 0.07 | 0.08 | 458 | 0.002 |
|  | Ca^2+^ | 0.32 | -1.93 | 466 | 0.016 |  | 0.04 | -1.19 | 462 | <0.001 |  |  |  |  | 0.866 |
|  | Mg^2+^ | 0.21 | -7.06 | 464 | 0.048 |  | 0.03 | -5.88 | 462 | <0.001 |  |  |  |  | 0.105 |
|  | pH | 0.27 | -8.03 | 508 | 0.027 |  | 0.09 | -9.68 | 516 | <0.001 |  |  |  |  | 0.297 |
| Leaf N | MAT | 0.63 | -0.39 | 27.1 | <0.001 |  | 0.13 | -0.41 | 27.1 | <0.001 |  |  |  |  | 0.521 |
|  | logMAP | 0.67 | -9.84 | 50.3 | <0.001 |  | 0.13 | -10.1 | 51.0 | <0.001 |  |  |  |  | 0.56 |
|  | AN |  |  |  | 0.167 |  | 0.02 | 0.010 | 19.4 | 0.004 |  | 0.08 | 0.012 | 23.1 | 0.001 |
|  | AP |  |  |  | 0.959 |  |  |  |  | 0.829 |  | 0.10 | 0.49 | 22.3 | <0.001 |
|  | AK | 0.24 | 0.04 | 17.3 | 0.039 |  | 0.06 | 0.035 | 17.0 | <0.001 |  | 0.07 | 0.015 | 22.2 | 0.002 |
|  | Ca^2+^ | 0.30 | 0.41 | 19.5 | 0.020 |  | 0.07 | 0.39 | 19.1 | <0.001 |  |  |  |  | 0.060 |
|  | Mg^2+^ | 0.39 | 1.95 | 19.3 | 0.007 |  | 0.08 | 2.10 | 18.9 | <0.001 |  | 0.06 | 0.87 | 22.9 | 0.006 |
|  | pH |  | 1.21 | 13.8 | 0.143 |  | 0.07 | 1.48 | 11.7 | <0.001 |  | 0.04 | -0.85 | 30.7 | 0.017 |
| Leaf P | MAT | 0.38 | -0.03 | 1.54 | 0.009 |  | 0.06 | -0.019 | 1.43 | <0.001 |  | 0.03 | -0.038 | 1.56 | 0.047 |
|  | logMAP | 0.34 | -0.64 | 3.03 | 0.014 |  | 0.05 | -0.430 | 2.43 | <0.001 |  | 0.05 | 1.57 | -3.09 | 0.013 |
|  | AN |  |  |  | 0.093 |  | 0.03 | 0.001 | 1.03 | 0.002 |  |  |  |  | 0.121 |
|  | AP |  |  |  | 0.215 |  | 0.02 | 0.018 | 1.03 | <0.001 |  | 0.03 | 0.021 | 1.07 | 0.038 |
|  | AK |  |  |  | 0.108 |  | 0.05 | 0.002 | 0.90 | <0.001 |  |  |  |  | 0.309 |
|  | Ca^2+^ | 0.45 | 0.03 | 0.989 | 0.035 |  | 0.04 | 0.020 | 1.04 | <0.001 |  |  |  |  | 0.643 |
|  | Mg^2+^ | 0.44 | 0.18 | 0.951 | 0.004 |  | 0.08 | 0.140 | 1.00 | <0.001 |  |  |  |  | 0.745 |
|  | pH |  |  |  | 0.362 |  | 0.01 | 0.054 | 0.802 | <0.001 |  |  |  |  | 0.627 |
| Leaf K | MAT |  |  |  | 0.112 |  |  |  |  | 0.245 |  |  |  |  | 0.543 |
|  | logMAP |  |  |  | 0.106 |  |  |  |  | 0.226 |  |  |  |  | 0.226 |
|  | AN |  |  |  | 0.589 |  |  |  |  | 0.975 |  |  |  |  | 0.984 |
|  | AP |  |  |  | 0.996 |  |  |  |  | 0.827 |  |  |  |  | 0.773 |
|  | AK |  |  |  | 0.368 |  |  |  |  | 0.827 |  |  |  |  | 0.684 |
|  | Ca^2+^ | 0.30 | 0.19 | 9.97 | 0.020 |  |  |  |  | 0.069 |  |  |  |  | 0.318 |
|  | Mg^2+^ |  |  |  | 0.151 |  |  |  |  | 0.302 |  |  |  |  | 0.405 |
|  | pH |  |  |  | 0.122 |  |  |  |  | 0.101 |  |  |  |  | 0.671 |
| Leaf Ca | MAT | 0.26 | -0.40 | 20.1 | 0.030 |  | 0.06 | -0.33 | 20.2 | <0.001 |  |  |  |  | 0.920 |
|  | logMAP |  |  |  | 0.087 |  | 0.04 | -6.01 | 33.2 | <0.001 |  | 0.04 | 17.4 | -36.9 | 0.021 |
|  | AN |  |  |  | 0.928 |  |  |  |  | 0.658 |  |  |  |  | 0.955 |
|  | AP |  |  |  | 0.825 |  |  |  |  | 0.655 |  |  |  |  | 0.605 |
|  | AK |  |  |  | 0.163 |  | 0.04 | 0.03 | 11.8 | 0.001 |  | 0.05 | -0.012 | 12.1 | 0.009 |
|  | Ca^2+^ |  |  |  | 0.228 |  |  |  |  | 0.186 |  |  |  |  | 0.318 |
|  | Mg^2+^ |  |  |  | 0.485 |  |  |  |  | 0.417 |  |  |  |  | 0.405 |
|  | pH | 0.46 | 3.24 | -6.29 | 0.003 |  | 0.13 | 3.06 | -3.98 | <0.001 |  |  |  |  | 0.671 |
| Leaf Mg | MAT |  |  |  | 0.100 |  | 0.02 | -0.036 | 3.527 | 0.007 |  |  |  |  | 0.862 |
|  | logMAP |  |  |  | 0.167 |  |  |  |  | 0.050 |  |  |  |  | 0.369 |
|  | AN |  |  |  | 0.541 |  |  |  |  | 0.566 |  |  |  |  | 0.713 |
|  | AP |  |  |  | 0.615 |  |  |  |  | 0.745 |  |  |  |  | 0.544 |
|  | AK |  |  |  | 0.240 |  |  |  |  | 0.081 |  |  |  |  | 0.514 |
|  | Ca^2+^ |  |  |  | 0.216 |  |  |  |  | 0.208 |  |  |  |  | 0.811 |
|  | Mg^2+^ |  |  |  | 0.255 |  |  |  |  | 0.217 |  |  |  |  | 0.933 |
|  | pH |  |  |  | 0.166 |  | 0.03 | 0.26 | 1.36 | <0.001 |  |  |  |  | 0.677 |

Sample sizes are the same as in Table 1. Abbreviations are as: MAT, mean annual temperature (℃); MAP, mean annual precipitation (mm); AK, soil available potassium of topsoil (0−0.30 m) (mg kg^-1^); AN, soil available nitrogen of topsoil (0−0.30 m) (mg kg^-1^); AP, soil available phosphorus of topsoil (0−0.30 m) (mg kg^-1^); Ca^2+^, soil exchangeable Ca^2+^ of topsoil (me 100g^-1^); Mg^2+^, soil exchangeable Mg^2+^ of topsoil (0−0.30 m) (me 100g^-1^); pH, soil pH value of topsoil (0−0.30 m).**TABLE S4∣** Pearson's correlations between PC1*_C_*_sCa_ and PC1_PK_ for the ecosystem, species-by-sites of three forest types and within two broadleaved species.

|  | Ecosystem | Species-by-sites | | |  | Within species | | | |
| --- | --- | --- | --- | --- | --- | --- | --- | --- | --- |
|  |  | EBF | MDEBF | DBF |  | | *Qw* | *Bp* |  |
| *R* | 0.43 | -0.19 | 0.14 | -0.13 |  | | 0.12 | 0.14 |  |
| *P* | 0.12 | 0.07 | 0.11 | 0.19 |  | | 0.11 | 0.35 |  |

PC1*_C_*_sCa_, the PC1 scores of leaf *C*_S_ and Ca concentrations; PC1_PK_, the PC1 scores of P, K and got PC1 scores of leaf P and K concentrations (PC1_PK_). These correlations are non-significant, with *P* > 0.05, demonstrating that these leaf structural traits are orthogonal to leaf metabolic ones. Abbreviations and sample sizes for each forest type and species are described in table 1.

**TABLE S5∣** Explained variances for the linear correlations between the PC1*_C_*_sCa_ and PC1_PK_ and leaf N, Mg concentrations from species to ecosystem.

|  |  |  | PC1*_C_*_sCa_ | PC1_PK_ |
| --- | --- | --- | --- | --- |
| Variance explained | | |  |  |
|  | Ecosystem | | 94% | 79% |
|  | Species-by-sites | EBF | 77% | 74% |
|  |  | DBF | 83% | 73% |
|  |  | MDEBF | 83% | 74% |
|  | Within species | Qw | 82% | 80% |
|  |  | Bp | 83% | 70% |
| Correlations with leaf N | | |  |  |
|  | Ecosystem | | 0.47 | 0.77^***^ |
|  | Species-by-sites | EBF | -0.06 | 0.12 |
|  |  | DBF | -0.30^***^ | 0.27^***^ |
|  |  | MDEBF | -0.17 | 0.58^***^ |
|  | Within species | *Qw* | 0.24^*^ | 0.54^***^ |
|  |  | *Bp* | -0.19 | 0.28 |
| Correlations with leaf Mg | | |  |  |
|  | Ecosystem | | 0.64^**^ | 0.61^*^ |
|  | Species-by-sites | EBF | -0.21 | 0.61^***^ |
|  |  | DBF | 0.49^***^ | 0.16 |
|  |  | MDEBF | 0.53^***^ | 0.23^*^ |
|  | Within species | *Qw* | -0.37^***^ | -0.11 |
|  |  | *Bp* | 0.49^***^ | 0.05 |

Abbreviations and sample sizes for each forest types and species are described in Tables 1 and S6. The statistical significance is indicated as: ^***^ ***P*** < 0.001; ^**^ 0.001 < ***P*** < 0.01; ^*^ 0.01 < ***P*** < 0.05.

**TABLE S6∣** Pearson's correlation coefficients (lower diagonal) and phylogenetically independent contrasts (upper diagonal) among six leaf functional traits for 20 woody dicots

|  | *Ca*_cellwall_ | *C*_cellwall_ | Protopectin | Lignin | *Ca*_leaf_ | *C*_leaf_ |
| --- | --- | --- | --- | --- | --- | --- |
| *Ca*_cellwall_ |  | -0.582^**^ | 0.758^***^ | -0.203 | 0.578^**^ | -0.752^***^ |
| *C*_cellwall_ | -0.783^***^ |  | -0.452 | 0.879^***^ | -0.183 | 0.503^*^ |
| Protopectin | 0.789^***^ | -0.782^***^ |  | -0.234 | 0.420 | -0.667^**^ |
| Lignin | -0.659^**^ | 0.878^***^ | -0.70^**^ |  | -0.178 | 0.567^**^ |
| *Ca*_leaf_ | 0.589^**^ | -0.30 | 0.458^*^ | -0.483^*^ |  | -0.709^***^ |
| *C*_leaf_ | -0.706^**^ | 0.635^*^ | -0.648^**^ | 0.715^**^ | -0.796^***^ |  |

^***^ ***P*** < 0.001; ^**^ 0.001 < ***P*** ≤ 0.01.

Abbreviations are described in table S3

**TABLE S7∣** Shapiro–Wilk's tests and phylogenetic signals for six leaf traits of 20 broadleaved woody dicots.

| Leaf functional traits | Unit | Mean | SE | CV | Shapiro–Wilk's test | Blomberg's *K* |
| --- | --- | --- | --- | --- | --- | --- |
| *Ca*_cellwall_ | mg g^-1^ | 18.2 | 1.92 | 47% | 0.96 | **0.82** |
| *C*_cellwall_ | mg g^-1^ | 510 | 6.17 | 5% | 0.95 | **0.91** |
| Protopectin | mg g^-1^ | 83.0 | 6.01 | 32% | 0.96 | **0.98** |
| Lignin | mg g^-1^ | 431 | 22.7 | 24% | 0.96 | 0.67 |
| *Ca*_leaf_ | mg g^-1^ | 15.6 | 1.77 | 51% | 0.95 | 0.78 |
| *C*_leaf_ | mg g^-1^ | 468 | 4.40 | 4% | 0.91 | **0.99** |

Mean values for all traits were calculated on the original scale. SE, standard error; CV, variation coefficient. *Ca*_cellwall_, calcium concentration in the cell wall; *C*_cellwall_, carbon concentration in the cell wall; Protopectin, protopectin concentration in the cell wall; Lignin, lignin concentration in the cell wall; *C*_leaf_, leaf carbon concentration; *Ca*_leaf_, leaf calcium concentration. All the data were normally distributed. Blomberg's *K* values in bold indicate they are significant at *P* < 0.05.
